# A framework for the detection and attribution of biodiversity change

**DOI:** 10.1098/rstb.2022.0182

**Published:** 2023-07-17

**Authors:** Andrew Gonzalez, Jonathan M. Chase, Mary I. O'Connor

**Affiliations:** ^1^ Department of Biology, McGill University, Montreal, Canada H3A 1B1; ^2^ Quebec Centre for Biodiversity Science, Montreal, Canada H3A 1B1; ^3^ German Centre for Integrative Biodiversity Research (iDiv) Halle-Jena-Leipzig, Leipzig 04103, Germany; ^4^ Institute of Computer Science, Martin Luther University Halle-Wittenberg, Halle (Saale) 06099, Germany; ^5^ Department of Zoology and Biodiversity Research Centre, University of British Columbia, Vancouver V6T 1Z4, Canada; ^6^ Santa Fe Institute, Santa Fe, NM 87501, USA

**Keywords:** causal inference, diversity, time series, monitoring, anthropocene, global change

## Abstract

The causes of biodiversity change are of great scientific interest and central to policy efforts aimed at meeting biodiversity targets. Changes in species diversity and high rates of compositional turnover have been reported worldwide. In many cases, trends in biodiversity are detected, but these trends are rarely causally attributed to possible drivers. A formal framework and guidelines for the detection and attribution of biodiversity change is needed. We propose an inferential framework to guide detection and attribution analyses, which identifies five steps—causal modelling, observation, estimation, detection and attribution—for robust attribution. This workflow provides evidence of biodiversity change in relation to hypothesized impacts of multiple potential drivers and can eliminate putative drivers from contention. The framework encourages a formal and reproducible statement of confidence about the role of drivers after robust methods for trend detection and attribution have been deployed. Confidence in trend attribution requires that data and analyses used in all steps of the framework follow best practices reducing uncertainty at each step. We illustrate these steps with examples. This framework could strengthen the bridge between biodiversity science and policy and support effective actions to halt biodiversity loss and the impacts this has on ecosystems.

This article is part of the theme issue ‘Detecting and attributing the causes of biodiversity change: needs, gaps and solutions’.

## 1. Introduction

Humans have transformed the processes generating and maintaining biodiversity from the smallest to the largest spatial scales of the biosphere. While the magnitudes and even directions of biodiversity change are varying from place to place, there is considerable evidence that multiple dimensions of biodiversity are changing rapidly in many places, including genetic diversity [1], population abundances [2], range sizes and distribution [3,4], turnover in community composition [5] and global species number [6]. We also know that local and regional diversity are changed by human and natural drivers, including land and sea use change, climate change, pollution, exploitation and invasive species [7] as well as many conservation activities designed to protect and restore biodiversity [8,9]. Human drivers interact and exert their influence at different spatial scales [10], and this makes the task of attributing trends in biodiversity to human causes across scales particularly challenging [11–14].

Formalizing how we update our understanding of how and why biodiversity is changing is vital if we are to track our progress to the goals and targets of the global biodiversity framework under the United Nations (UN) Convention for Biological Diversity and UN Sustainable Development Goals. To date, our knowledge of the pace and direction of biodiversity change has accumulated from synthesis assessments of many relevant studies [15]. For example, statements that the current rate of extinction is 10–100 times the background rate in the fossil record involves the detection of a significant increase in estimated extinction rate [16]. However, this estimate has high uncertainty because of incomplete knowledge of past and current extinction rates estimates for known and unknown taxa [16,17]. Estimates of extinction rates are complemented by analyses mapping biogeographic trends in biodiversity with large datasets synthesizing time series from long-term experimental sites or systematic surveys. These analyses are revising our understanding of biodiversity change and its impacts on ecosystems at different scales [1,5,12,18–21] and the geographically variable influence of human drivers [22]. However, these studies adopt different criteria and statistical procedures for detecting biodiversity change and understanding its impacts. A literature composed of inferences based on different criteria makes robust assessments difficult because uncertainty in the attribution process cannot easily be aggregated across studies. A framework is needed to support a common approach to assessing and understanding biodiversity trends that produces updated statements of confidence in their attribution over time [19,23].

**Figure 1.**
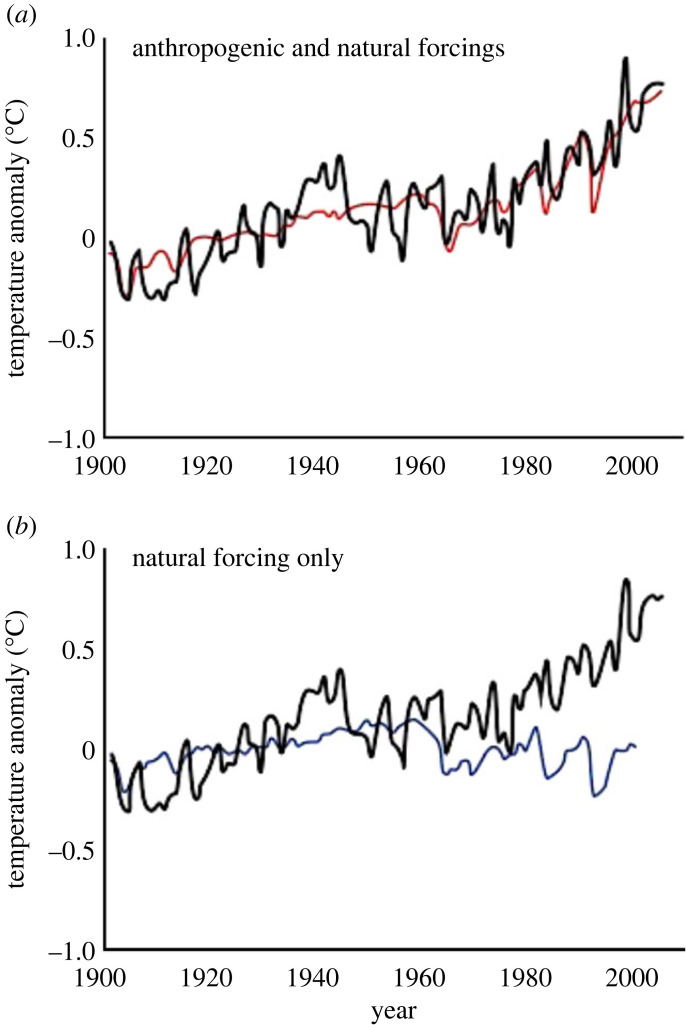
Example of detection and attribution in climate science, in the form of detection of trends in global temperature, and attribution to human versus natural factors using comparison of observations (anomalies) with different ways of modelling temperature that do (*a*), or do not (*b*) include human-driven elevation of greenhouse gas concentrations and related forcing [35]. This detection and attribution analysis and figures representing it like this one proved highly influential in the process of accepting the inference that climate change in the present era is generally anthropogenic in nature. (Online version in colour.)

In climate science, a *detection and attribution* framework has been developed under the auspices of the Intergovernmental Panel on Climate Change (IPCC) [24,25]. In this context, the first objective is to assess the evidence that some aspect of climate (e.g. extreme weather [26]), and/or a system affected by climate (e.g. ocean chemistry as indicated by pH), has changed over time (detection). The second objective is then to evaluate the contributions of multiple potential drivers of this change (attribution). The climate detection and attribution framework defines climate change in an explicitly statistical sense using scientific observations and measurements (e.g. the Keeling curve of CO_2_), inference methods, and criteria for evaluating uncertainty at different scales (global, regional, etc.; [25]). This exercise led to highly influential knowledge products such as the robust attribution of global temperature change to natural versus anthropogenic factors (figure 1). O'Connor *et al.* [28] outlined how to set criteria for data, statistical analysis and inference about detection and attribution for climate change impacts, and these standards may also be useful for biodiversity change detection and attribution.

**Figure 2.**
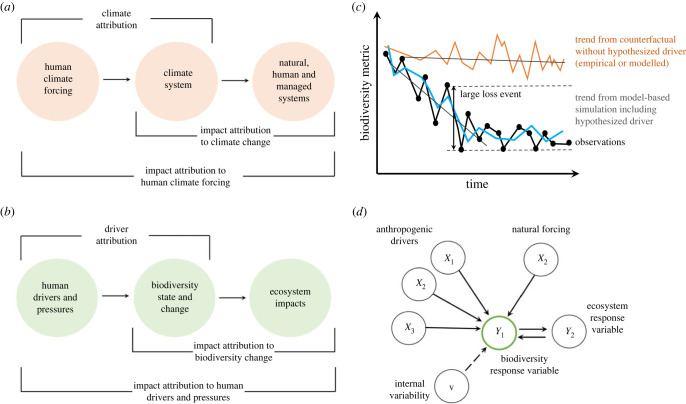
(*a*) The relationships among the systems involved in climate detection and attribution (after [27]). Climate attribution as the identification of the causal links between human climate forcing via greenhouse gases emissions as the explanation for climate warming, impact attribution as the attribution of climate impacts on natural, human and managed systems. (*b*) Human driver attribution as the identification of the causal links explaining how human drivers and pressures result in altered patterns of biodiversity change, and the consequences this has for natural and managed ecosystems. (*c*) Attribution of human pressures as causes of observed biodiversity change (black line) requires a model including the putative human factor (blue line), and a comparison with a counterfactual series (obtained from a model or observations from reference sites) showing how biodiversity would change in the absence of the putative driver. Attribution can focus on trends in the mean, variance and fluctuation event sizes. (*d*) The detection and attribution framework for biodiversity change places biodiversity variables at the centre of the analysis. Conceptual models are formalized as causal (directed) graphs identifying the direct and indirect effects of driver and pressure variables (*X_n_*) on the biodiversity response variable (*Y*_1_, green). The dotted arrow indicates dependency owing to an unobserved latent variable (*v*) that may represent the eco-evolutionary processes governing internal variability of the focal biodiversity response variable. (Online version in colour.)

Here, we introduce a detection and attribution framework designed explicitly for biodiversity change and its ecosystem impacts (figure 2*b*). We are motivated to define the features of this framework because of the focus on changing trends under the UN Convention on Biological Diversity's Global Biodiversity Framework (GBF). Realization of the GBF goals and 2030 targets will require rapid detection of improvements in biodiversity and ecosystem trends coupled with assessments of changing driver impacts where conservation action has succeeded or failed [29].

The framework for the detection and attribution of temporal change in biodiversity is based on clear statements of causality, based on alternative models and hypotheses, the best methods for observing and quantifying biodiversity, and statistical methods to detect and attribute change. We link five steps—causal modelling, observation, estimation, detection and attribution—to form an iterative cycle of inference that produces a clear and regularly updated statement of confidence in the reported outcomes. An important feature of this framework is the need to ensure standards that maximize confidence as information is transferred from step to step and guide analytical choices away from those that produce low confidence. We close by outlining how a framework for detecting biodiversity change with an objective of attribution for policy action should guide monitoring to provide data as input into biodiversity models and scenario-based projections of future outcomes for biodiversity and ecosystem change impacts on the economy and human wellbeing [30].

## 2. Defining key concepts for detection and attribution in biodiversity science

We start by defining the key concepts—detection and attribution—for biodiversity change by adapting existing definitions used in climate and Earth system science provided by [25]. For a biodiversity framework, these terms would be defined as below.

**Detection**: the process of demonstrating that a measure of biodiversity has changed relative to a baseline or reference distribution characterising undisturbed variation (counterfactual state), or an appropriate model-derived null expectation of biodiversity change in the absence of a human driver(s). A clear statement of statistical confidence should be given.

Detecting temporal change in biodiversity is the process of distinguishing change over time from an alternative hypothesis of no change over time (figure 2*c*). Detecting change therefore requires distinguishing a real trend reflecting historical influence or systemic change by some causal driver or drivers to be determined in an attribution process from a spurious trend, which might be owing to measurement or estimation error or process error.

Crucial to the detection step is the form of change to be detected (see box 1). This can be a linear or nonlinear trend in the mean, or a shift in the variance, or other description of the variability of a time series (periodicity, autocorrelation). Alternatively, we may detect a one-off ecological event that is highly circumscribed in time but may represent a significant loss or gain of biodiversity and impact on ecosystem processes and services.

Box 1. **Biodiversity fluctuations: the backdrop for detecting and attributing human influence**

Biological diversity is a measure of life's compositional variation across different levels of organization—genome, population, species, and ecosystems—and its changing state over dimensions of space and time [31,32]. Irrespective of the metric we chose to quantify biodiversity (i.e. richness, entropy-based measures of diversity, turnover) our aim is to observe and detect temporal change (figure, panel (*a*)). With a reliable observation processes we may see sustained (stationary) fluctuations in the metric but no systematic change (trend) in the statistics describing those fluctuations in the time series (mean, variance or autocorrelation); by contrast, non-stationary fluctuations may involve systematic trends in these statistical moments over time and space. These shifts in the statistics of the biodiversity metric may be visualized by changes in the distributions describing these fluctuations (figure, panel (*b*)). A comparison with a reference distribution, either from a historical pre-perturbation period, or from a contemporary unperturbed reference site (a counterfactual condition) is needed to assess change.

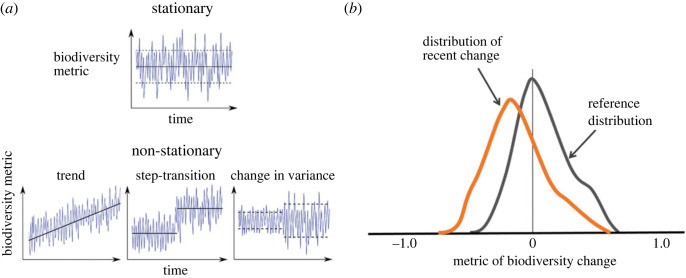

Biodiversity *change* is inherently scale dependent in space and time [33]. A rate of change detected at one scale may not be representative of the rate of change at another scale. For this reason, biodiversity change may not be ergodic (i.e. where averages over time equal averages over space); biodiversity change detected from a single, or small number of, short time series, may not capture the spatio-temporal dynamics of biodiversity change over large spatial extents. Scale explicit analyses are needed resolve contrasting trends in biodiversity change [11,12].

It is against this highly dynamic backdrop of change that we must detect the signal of human impacts on biodiversity change. Change in human drivers is also scale-dependent, so an attribution of causes for patterns of biodiversity change at one scale may not lead to the same conclusion if the attribution is conducted at another scale. For example, the impacts of habitat loss and fragmentation on biodiversity may be great at the scale of local habitat patches, but not apparent at landscape or broader scales until levels of habitat loss pass a threshold of fragmentation. As a result, detection and attribution should involve a joint analysis of biodiversity (gains and losses) and driver variables, and involve a clear statement of confidence, an explicit reference to the dimension of scale in space and time.

Usually, a comparison of a measure of biodiversity change is made with respect to a reference state such as a historical baseline, or to a spatial reference state if historical baselines are unavailable or inappropriate (e.g. if the system is undergoing directional change). We will return to this issue of detecting change below.

**Attribution**: the process of evaluating the relative contributions of multiple potentially causal factors to detected biodiversity change with an assignment of statistical confidence to the causal models used to estimate these effects.

Attribution is the identification of cause-and-effect relationships between hypothesized drivers and changes in biodiversity variables. By cause, we mean that *X* causes *Y* if a perturbation in a driver variable *X* can result in a change in future values of a biodiversity variable *Y* [34]. Causality in complex systems is assumed to be probabilistic, in which case we say *X* causes *Y*, if a change in *X* causes a change in the probability distribution of biodiversity variable *Y* (box 1). Crucially, we do not require that *Y* responds to *X* in the same manner (e.g. magnitude, direction) in all contexts to identify *X* as the cause of *Y*.

We can attribute a detected change in a specific causal factor *X* if the pattern of change in *Y* is consistent with a statistical and/or process-based model that includes *X* and is *inconsistent* with a model that is otherwise identical but excludes *X* (counterfactual case) (figure 2*c*, or figure 1*b* for global average temperature trends). Attribution in coupled human and natural systems [36] may involve accounting for multiple variables, including those with direct and indirect causal effects that may have lags in time and over space. The relationship among the variables is typically shown as a causal graph (figure 2*d*). Attribution must also account for the inherent variability in describing the probability distribution for the focal biodiversity variable, *Y*.

For biodiversity change detection and attribution, two other key concepts are necessary—observation and estimation.

**Observation:** the process of recording elements that represent aspects of biodiversity (e.g. genes, species, traits, etc.) using methods of recording that ascribe to scientific principles of reproducibility, replication, and objectivity.

Observations can range from one-time observation events with unique geospatial coordinates to repeated time series from experiments or systematic surveys from unmanipulated ecosystems [37]. Efforts are underway to establish standards (e.g. essential variables) for the capture of biological inventories for biodiversity monitoring, modelling and assessment [38,39]. Ideally, observations of biodiversity should be accompanied by observations of human impacts and other environmental factors hypothesized to be causally linked to biodiversity change to allow a stronger attribution analysis downstream.

**Estimation:** the process of combining observations into statistical estimates of metrics quantifying aspects of biodiversity, including the use of measures of uncertainty and replication.

The methods of biodiversity estimation have a long and rich history [40–42]. The detection and attribution framework we outline below builds on this history and focuses on the generation of biodiversity observations and estimates of metrics, in particular with regard to the unique nature of estimating biodiversity change with incomplete observations [43]. We focus our discussion below on the particularities of applying the detection and attribution framework to biodiversity datasets.

## 3. A detection and attribution framework for biodiversity change

A detection and attribution framework for biodiversity change is a formalized interlinked process for model-based detection of change and causal inference based on iterative data collection and monitoring. It must be built on best practices for collecting data via biodiversity observation and measurement [37]. However, it is not possible to complete the detection and attribution process with data alone; there needs to be a clear and intentional consideration of inference and the contrasting model(s) upon which the inference is made. This is because we cannot make strong inferences without making assumptions, so that grounding these assumptions in alternative formal models allows us to make these assumptions clear. The confidence we have in the final statement will also depend on the accurate propagation of uncertainty along the chain of steps.

**Figure 3.**
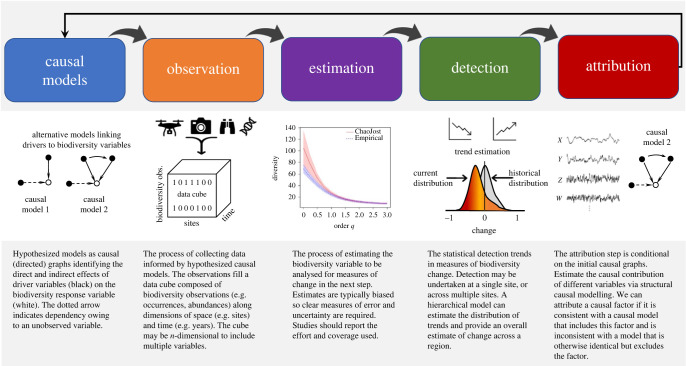
The five steps in the detection and attribution workflow. The process begins with causal models of our understanding of biodiversity change, which in turn guide the work of observation, estimation of essential biodiversity variables and their use in the detection and attribution steps. Information generally flows from left to right, but the workflow is repeated iteratively as new data are collected, technologies are deployed, and our confidence in the methods used to detect and attribute causes is improved. Increases in confidence will arise from observations and adaptive monitoring that are designed and coordinated to detect change and reduce uncertainty in the attribution of human drivers as causes for trends. (Online version in colour.)

The framework has five steps (figure 3). It describes an analytical workflow that involves a rigorous transfer of information across the steps needed to reliably detect change and achieve strong inference for causal attribution.

### (a) Step 1: causal models

The causal structure of an ecosystem is assessed by performing a physical intervention on one or more variables (e.g. manipulating precipitation, pollution or species addition or removal), while observing the response of other variables over time. However, in many cases relevant to biodiversity change assessments, experiments and related interventions are unethical, expensive, or impossible to realize and so we must rely on observational data from repeated surveys at one or more sites. Reference sites with and without a history of relevant drivers can provide valuable causal understanding. Recent developments in causal discovery and inference open the door to the estimation of alternative causal models needed for detection and attribution [44–49].

#### (i) Defining hypotheses

The framework begins by defining hypotheses and assumptions that will guide the detection and attribution workflow. This step draws upon general scientific understanding from the relevant literature on the focal biodiversity variable(s) and drivers. For example, observations, theory and experiments guide clear hypotheses for how habitat loss and fragmentation can change biodiversity, and this understanding has been used to formally frame detection and attribution research [50,51].

#### (ii) From hypothesis to causal models

We advocate applying the principles of causal analysis and causal networks as the basis for the analyses in the next steps of the framework [44,52,53]. A causal network diagram (i.e. a directed acyclic graph (DAG); [53]; figure 2*d*) describes the relationship between the putative causal factors hypothesized to drive biodiversity change. It can be seen as a non-parametric structural equation model that explicitly lays out directed paths between variables, but the form of the relationship between two variables does not matter, only its direction. The rules underpinning DAGs are consistent whether the relationship is linear or more complicated. A causal analysis is the logical approach to assessing the influence of different direct and indirect relationships among the multiple potential interacting factors, typically a suite of natural and human drivers. Causal analyses can be a simple logical structure of relationships, or a fully dynamic Bayesian network model.

Potential causal links in a model can be derived from expert knowledge and findings from controlled experiments and theory (step 1b). When we lack a model for causal inference, we can apply methods for causal discovery [54,55]. Methods and algorithms for causal discovery derive causal knowledge in the form of a DAG directly from patterns in the observational data and require time series of the response and predictor variables. These methods, which also make assumptions, can provide understanding of the relationships among the variables causing change in biodiversity owing to human drivers (e.g. [36,44,53]). In climate science, these causal graph models are called ‘causal fingerprints’ that are learned from multivariate climate datasets [56–58]. A similar methodology may be applied to the identification of causal fingerprints for biodiversity change, assuming the properties of the available time series (e.g. stationarity) meet the assumptions of the algorithm used to identify the causal graph (e.g. causal sufficiency that assumes that all variables are observed versus the case where we suspect unobserved latent variables are involved [59]).

An alternative approach is to build process-based models that encode ecological processes as coupled (stochastic) equations [60]. These models are mechanistic in the sense that parameters correspond to states and rate processes linked to measurable constraints (e.g. trait-based functional responses to the environment) that interact to govern the dynamical state of the system. An example would be consumer–resource systems of equations typically used to model ecological dynamics [61]. A process model can be fitted to time series data with and without potential causal drivers to support detection and attribution [62]. When such parametric dynamical models cannot be built, agent-based models may be used for causal inference [63]. When formal causal models are not readily available, verbal or graphical expressions of directed causal relationships are valuable, and in fact a necessary starting point in this framework.

We recommend strong hypothesis tests by comparing alternative causal network models (fingerprints) to make the strongest inference possible for attribution in step 4 below [44,53]. Knowing this, the collection of observations in step 2 should be guided by the hypothesized causal dependence between the drivers and biodiversity variables formalized by the graphical models used for attribution.

### (b) Step 2: observation

Given the framework's emphasis on biodiversity change, the relevant observation process is on time-ordered sequences of biodiversity observations. Modern biodiversity science has an array of methods and technologies for observing individuals and their state (their genotype, taxonomic identity, functional form and traits comprising their phenotypes), and the diversity and composition of the assemblages they make up. Observation and subsequent steps in this framework can involve a combination of observations using different methods, as long as uncertainties are accounted for [37].

Observations are almost always incomplete samples of biodiversity in nature, that when combined translate into incomplete representations of biodiversity change over time and space [43,64,65]. Difference in the detectability of species by different methods and instruments is a key consideration and potential source of bias in this step that can intervene in subsequent steps [66]. Observations of a particular place or ecological group are stored as a site × variable table (e.g. a site × abundances or site × species), which when ordered by time fills a multi-dimensional data cube (site × variable × time; [67]) or hypercube when there are more than three dimensions [68]. If the table is incomplete, gaps can be completed with estimates made in step 3.

Observations made or used *post hoc* in the context of this framework are placed in an analytical framework that requires an estimate of uncertainty (are differences among observations real, or rather owing to methodological differences or sampling error?). Other aspects of observations such as the choice of sites to be monitored, and the duration, frequency and coverage of effort mobilized for the observation step are crucial to the strength of the inferences that can be made in subsequent steps (estimation, detection and attribution). Recent work by Zhang and colleagues [69] revealed an important bias whereby high levels of habitat change led to the cessation of bird sampling in biodiversity time series, resulting in a potential underestimate of biodiversity loss; survey routes that were continued despite major habitat changes were more likely to experience reduced diversity.

For strongest inference at the end of the detection and attribution process, we recommend prioritizing observations based on the requirements for estimating (step 3) essential biodiversity and ecosystem variables (EBVs; [70,71]), that can be shared easily across studies and used in multiple workflows for subsequent modelling and comparison. EBV workflows offer methodological standards for making observations and providing them in usable open data forms [72]. The joint observation of drivers (e.g. land cover, climate variables) that are hypothesized to be important for the attribution step (step 5) with data that are downscaled to the relevant scales of the focal organisms and assemblages should also be made.

### (c) Step 3: estimation

Estimation is the process of calculating a biodiversity metric from an incomplete sample (the observation or set of observations), with the aim of reducing the influence of sampling bias and imperfect detection of species. There are multiple metrics used to capture different dimensions of biodiversity [70], which are associated with different biases and other forms of uncertainty [73]. The accuracy and errors associated with different metrics also inherently depend on the scale and spatial resolution of observation [65].

The choice of metric for estimation, its scale dependence and sensitivity to bias or uncertainty can strongly influence interpretation of biodiversity change in steps 4 and 5 below [74–76]. Humans directly and indirectly impact different biodiversity variables by changing the genetic structure of populations, the number of species and their relative abundances, and the composition of the species in the assemblage [77,78]. Measures for changing species composition via species turnover may be more sensitive to human drivers than estimates of the total number of species, owing both to constraints (e.g. resources) on species richness in changing environments [31,79], as well as the insensitivity of this estimator to changes in harder-to-detect rare species [76,80].

Some metrics provide more reliable (less biased) estimates of biodiversity and its change than others [76,81]. Lande *et al*. [82] suggest the metric chosen should be non-parametric, applicable to any community, statistically accurate, with small bias and variance in samples of moderate size. A decision must be made early in the detection and attribution workflow to choose the relative sensitivity of the diversity metric towards rare or common species, although it is generally preferable to use multiple metrics [73]. For example, the most common metrics, and their effective number equivalents [83] are species richness, variants of Shannon information and Simpson concentration. Each index reveals different dimensions of biodiversity and its change, but also have different biases; some better capture change in rare species (e.g. species richness), abundant species (e.g. Shannon) or evenness (e.g. Simpson, or 1-Simpson, which is the probability of interspecific encounter). Ultimately, they can all be derived as particular cases of a general entropy [84,85] which provides a rigorous basis for comparison.

Hierarchical Bayesian multispecies site-occupancy and distribution models have been developed [86,87] to improve the estimate of diversity in landscapes from an incompletely observed species × site incidence matrix (obtained in step 2). These models compute unbiased estimates of site-specific and total species richness, species-specific site occupancy and similarity between sites and between species, and allow covariates to be introduced, while accounting for parameter uncertainty. These hierarchical Bayesian models require that survey sites are distributed randomly or are an otherwise representative sample of the greater area about which inference is required. Otherwise, inference is restricted to the quadrats sampled.

### (d) Step 4: detection

In this step the detection of biodiversity change is made relative to a baseline or reference distribution characterizing a relatively undisturbed site (a counterfactual state, or system), or an appropriate model-derived null expectation of biodiversity change in the absence of a human driver(s) (figure 2*c*).

The biodiversity time series generated from datasets produced in steps 2 and 3 provide the basis for detecting temporal change in biodiversity metrics. Trends can be assessed in univariate analysis of biodiversity metrics, such as estimated species richness (e.g. [81]), or via direct multivariate time series analysis of trends in multispecies assemblages, where trends in species' (or functional group) abundances are assessed within and across assemblages often using a generalized or Bayesian mixed model framework accounting for random effects of time, survey sites, autocorrelation and hierarchical errors (e.g. [77,88]).

The statistical procedures we choose should reflect key aspects of the causal model (step 1), such as whether we have hypothesized linear or nonlinear trends in the mean of the metric, or in other statistical moments, such as the variance or autocorrelation. Depending on the number of observations and the estimation procedures available, there are many options for statistical tests to detect trends and distinguish biodiversity change from the alternate hypothesis of no change [89].

Time series length is well known to influence trend detection. Short time series are often statistically underpowered and so false conclusions about the presence or absence of change (e.g. trends in mean or variance) may arise [81,90]. Longer time series tend to provide more reliable estimates of the trends detected and this will have consequences for the attribution conducted in step 5. The power to detect long-term trends in multispecies occupancy depends on whether the area covered by survey sites effectively cover species’ ranges and regions experiencing trends in occupancy [91].

Statistical power analysis provides a framework for assessing our ability to detect a certain magnitude of biodiversity change given the level of variability in the time series data and under a null hypothesis or an informed prior expectation of change [92,93]. Southwell *et al*. [93] found that the power to detect trends in species occupancy is sensitive to the direction and magnitude of the change in occupancy, detectability, initial occupancy levels, and the rarity of species. The statistical power required to detect a change of a given effect size will also vary with the statistical analyses used, and so it is advisable to compare multiple analyses to assess confidence in each result.

Historical baselines are needed to estimate the full extent of change in biodiversity over time, but limited data availability at appropriate time scales is a barrier. Mihoub *et al.* [94] reported most biodiversity monitoring schemes in Europe were initiated late in the twentieth century, well after anthropogenic pressures had already reached half of their current magnitude. Temporal baselines set long after the inception of biodiversity change will therefore underestimate the full range of impacts of anthropogenic drivers [19]. In addition, unbalanced datasets in terms of taxa and organization levels provide a biased understanding of biodiversity change over time. In the absence of adequate historical baselines, change can be assessed by comparing impacted sites with contemporary reference sites where putative drivers are absent [95].

### (e) Step 5: attribution

Attribution is the process of evaluating the relative contributions of causal factors explaining detected biodiversity change with an assignment of statistical confidence. In this step, the goal is to estimate the causal effects of one or more drivers on the focal biodiversity metrics via the causal models defined in step 1. This workflow leads to attribution of biodiversity change to one or more specific causal drivers if the observed trend is consistent with the prediction of the causal statistical model where the drivers cause a change in the probability distribution of the focal biodiversity variable. Alternatively, the observed trends is consistent with a process-based model including the hypothesized driver and is inconsistent with a counterfactual case that is otherwise identical but excludes the factor [96,97]. When a system model is absent, the attribution step can begin by assessing the relative explanatory power of informal models put down in step 1.

Systematic biodiversity change may involve long-term change in both anthropogenic (e.g. ongoing climate change, nutrient deposition) and natural drivers (e.g. successional change, changes in fire regimes). Models may inform the impacts of human drivers acting alone or as complexes involving compound effects [10,98,99]. In most cases, the strength and influence of these drivers will vary through time and over space, potentially involving feedbacks [100] and causal drivers may be geographically distant from the site where biodiversity change is observed (i.e. telecoupled effects, [57]).

Three key ingredients of attribution are required: evidence of consistency, evidence of inconsistency, and a statement of confidence [27].

*Evidence of consistency* requires that the detected change in biodiversity is consistent with the assumed driver(s) of change. For example, if we detect a change in species richness in a habitat fragment and we hypothesize this is owing to habitat isolation, then we must show that the effects owing to isolation translates into the observed change in richness (e.g. owing to loss of dispersal connectivity; [101]). The strength of our evidence of consistency is based on our ability to demonstrate the sign and magnitude of the relationship between cause and effect. Meta-analyses and other large scale syntheses provide a valuable means of demonstrating the strength of existing evidence for causal relationships across study systems.

*Evidence of inconsistency* reveals that the detected change in diversity is inconsistent with changes owing to alternative drivers. If more than one driver of change is acting on a community, and because we only observe the integral response of the community to all acting drivers, attribution requires evidence that the observed change has not been caused by alternative drivers. Evidence of inconsistency is necessary to avoid confirmation bias (i.e. the tendency to favour information that confirms existing preconceptions or hypotheses). Similarly, we want to avoid biased interpretation (i.e. where some hypotheses are not confronted with high standards of evidence).

Several methods exist for quantifying the strength of the causal links among variables that go beyond conventional regression models and explicitly include counterfactuals to assess the consistency (inconsistency) of the observed effect with the variables included (not included). We point the reader to several recent reviews of causal analysis for time series that use different definitions of cause and different assumptions and criteria for assigning it [34,44,45,96,102].

*A statement of confidence* in the attribution step is essential because we are always dealing with uncertainties associated with limited observational data from step 1 (i.e. samples, monitoring sites, studies in a meta-analysis), limited information about drivers (observation), imperfect correction for bias in diversity estimators, and uncertainty in our understanding of the nature of the ecological system (i.e. trophic structure), and uncertainties inherent to our modelling tools used to make inferences. A statement of confidence forces us to acknowledge these limits and quantifies the strength of our attribution statement in terms of a likelihood statement regarding how likely the change is caused by a given driver or set of drivers. The language adopted by the IPCC to convey measures of certainty expressed probabilistically (e.g. from virtually certain at greater than 99% probability, to exceptionally unlikely at less than 1%) could be used here to provide clarity for policymakers and consistency across UN conventions [103].

*Single step* versus *multi-step attribution:* attribution can be done in a single step or involve multiple steps of modelling and hypothesis testing. In the single step approach to attribution, one analysis can achieve full detection and attribution. The likelihood of detected biodiversity change is established from a model incorporating known causal drivers of change (step 1). This approach can be extended to multiple sites if time series are available across a network of sites. Hegerl *et al*. [25] introduced the idea of multi-step attribution when the conditions for single step attribution cannot be met. The multi-step approach includes first obtaining robust estimates of biodiversity change (detection), and then second, obtaining predictor drivers—either directly measured (from a designed survey or experiment) or derived from land use/climate change models. Then statistical models are used to combine predictors and estimates of biodiversity change to assess the likelihood that the trend found in the first step is explained by these drivers (e.g. [104]). Care is required to reduce the propagation of uncertainty across steps in the multi-step approach.

## 4. Examples

Elements of the detection and attribution process in biodiversity change research are not new, and many studies, syntheses and assessments have carried out at least part of the process we define. The best examples of detection and attribution inference will meet all criteria in each step outlined above, which includes the explicit consideration of uncertainties. Some attempts are more robust than others [28].

The attribution of habitat fragmentation to elevated rates and scales of biodiversity change is an area of current focus [50,51] and debate [105]. Boulinier *et al*. [78,106] provide a good example of an analysis detecting and attributing change in North American breeding bird assemblages owing to habitat fragmentation. They found that fragmentation was associated with higher temporal variability in community composition as a result of higher local extinction/colonization rates. They presented a clear verbal causal model (step 1) for biodiversity patterns and temporal change in breeding birds that informs the choice of sampling method (step 2), estimator (step 3), detection analysis (step 4) and partial attribution (step 5). Consistent with the causal model, which was based on habitat patch size, multiple scales of analysis were considered, and species were analysed in two groups: area-sensitive species (i.e. those most likely to change in diversity over time in fragmented landscapes) and non-area-sensitive species. The observation method was the breeding bird survey, for which sampling methods are understood and uncertainties can be quantified. The estimation method involved an EBV (species site occupancy) to estimate species richness while accounting for observation error. For the detection of change analysis, the authors used two parameters suited to the purpose: the rate of species' local extinction and the rate of species local turnover. These were based on the community analyses and on the estimation methods in the previous step. Missing from this analysis relative to the framework we outline is a statistical causal model and estimate; their analysis linear modes (regression and ANCOVA) to relate variables. Also missing were several elements of the attribution step, including consideration of alternate drivers of change over the period, or potentially confounding factors such as uncertainties in estimation of fragmentation and how it changed over time. Noting the limitations allows a more complete understanding of how we might further synthesize knowledge gained from detection and attribution exercises, especially when most studies can only report part of the process.

There are many other good examples in the literature to draw from that are clear on key elements of this framework. An example of a single step attribution analysis is by Harrison *et al.* [107] who show that trends in grassland species richness could be attributed to changing patterns of precipitation using a clear causal verbal model, standardized observation and estimation methods over time, and linear mixed effect models that allowed the assessment of alternate climate predictors. Millette *et al.* [1] use a multi-step approach with generalized additive models to attribute change in genetic diversity over space and time to human drivers (land use intensity, human population density). Elahi *et al.* [108] detect increases in marine species richness over time and were able to rule out certain potential drivers of biodiversity change through attribution using a model comparison approach. Daskalova *et al.* [109] used a multi-step attribution approach to assess trends in population change in the Living Planet Database. Chang *et al*. [47] applied a causal network approach (convergent cross mapping) to reveal that climate warming destabilizes plankton dynamics. We expect the strong causal inference framework for detection and attribution we have proposed will lead to a deeper understanding of the direct and indirect effects of multiple human drivers on biodiversity and other ecological impacts.

## 5. Next steps

The detection and attribution framework for biodiversity change outlines a workflow that has the potential to produce inferences about biodiversity change that are more transparent in their limitations and therefore potentially more robust than is possible in the *ad hoc*, *post hoc* and piecemeal fashion that is more common in the absence of the framework. These benefits are possible because the framework centres on strong inference for detection of change and attribution of that change to drivers. As a result, users can assess how best to design data syntheses, assessments or monitoring to minimize uncertainty and bias in detection and attribution that could arise upstream in steps 1–3.

The framework can guide the research and assessment process in a variety of ways and has the potential to bridge knowledge gains across disparate areas of biodiversity science. Research is still required within each step and for the transfer of information and uncertainty across steps. Developments in causal analysis are particularly relevant and can be coupled with ongoing developments in ecological theory [46]. Controlled experimental tests of theory provide a firm knowledge base to reinforce our confidence in the causal models formulated for step 1. Multi-causal models defining the relationships between biodiversity change and multiple interacting drivers at different scales are particularly challenging. Technological and theoretical advances are now linking remote observations to estimates of biodiversity change (e.g. for plant species [110–112]). Extensive effort is required to scale-up observations of biodiversity change at specific sites to broader regions [112] and reconstruct historical observations.

New biodiversity indices are being developed to quantify the link between the changing state of biodiversity and the resulting impacts of these changes on ecosystem benefits to people. For example, Soto-Navarro *et al*. [113] proposed the multidimensional biodiversity index designed to support national application and decision making for the Sustainable Development Goals. This addresses the common use of several unidimensional measures of biodiversity that are disconnected from ecosystem and societal outcomes. New research and innovation are needed in the estimation step for these indices and to deepen our understanding of the sampling and monitoring strategies that lead to sustainable collection of the time series they will need.

As has been done with climate change and its impacts, we can extend the detection and attribution workflow to the ecosystem *impacts* arising from the observed change in biodiversity (figure 2*b*). For example, we can estimate how changes in the rate of compositional turnover, or the rate of species loss, impacts measures of ecosystem functioning and stability, and how in turn they influence ecosystem services that determine outcomes for people and their livelihoods [20,100,114–116]. Advances in monitoring ecosystem services following the detection and attribution framework are needed [117]. The cumulative knowledge from these studies will form the basis for robust multilevel detection and attribution assessments in the field. This need has added importance as increasing numbers of businesses and companies in the financial sector are reporting their biodiversity impacts, assessing the risks arising from biodiversity loss and looking for means to diminish and offset their impacts [118].

## 6. Embedding the detection and attribution framework into systematic biodiversity monitoring and assessments of progress to international targets

**Figure 4.**
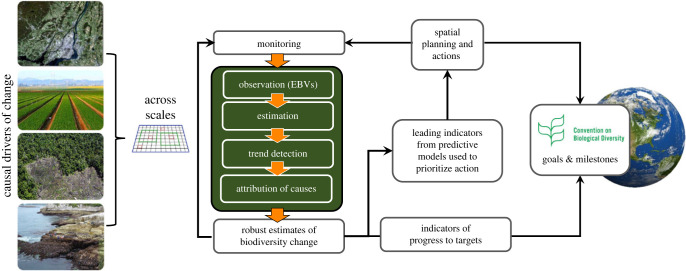
Embedding the detection and attribution framework into the cycle of monitoring that is required to assess and report progress toward the targets of the UN's Global Biodiversity Framework. Systematic detection and attribution according to the framework will increase confidence in the trends conveyed by the indicators for the targets, improve confidence in the models used to make forecasts of biodiversity change, guide planning and assess the outcomes of conservation action at local, national and global levels.(Online version in colour.)

Systematic monitoring for biodiversity change is often designed to detect trends up to a certain level of statistical power, but few are designed with detection and attribution objectives linked to specific biodiversity targets [119]. By embedding the detection and attribution framework in an iterative monitoring cycle, we expect improved confidence in the causes of the detected biodiversity trends that can be used to guide more effective conservation actions and long-term planning [120] (figure 4). This should spur the widespread adoption of a detection and attribution framework to guide monitoring that is required to assess and rapidly update our knowledge of how conservation action is leading to progress towards the Convention on Biological Diversity's post-2020 Global Biodiversity Framework.

### (a) Detection and attribution for biodiversity assessments

Biodiversity assessments are conducted via an extensive expert-based synthesis of the primary literature, such as the assessment reports conducted by the Intergovernmental Science-Policy Platform on Biodiversity and Ecosystem Services (IPBES; e.g. IPBES 2019, the global assessment report). The climate research community has seen the benefits of the integration of detection and attribution into its assessment process as it has sought to attribute extreme weather and climate events to human caused climate change. A quote from Stott *et al*. [121, p. 310] is relevant here:

the overarching challenge for the community is to move beyond research-mode case studies and to develop systems that can deliver regular, reliable and timely assessments in the aftermath of notable weather and climate-related events, typically in the weeks or months following (and not many years later as is the case with some research-mode studies).

This quote holds for biodiversity science if we replace ‘notable weather and climate-related events' with ‘notable ecological events’. We see a future where the systematic application of a detection and attribution framework for biodiversity supports the rapid attribution of causes for ecological or ecosystem impacts arising from large magnitude changes in biodiversity (gains or losses).

## 7. Conclusion

We have outlined a framework for detecting and attributing biodiversity change. The challenge of understanding the nature and causes of biodiversity change is one of the greatest facing science and society, and clear robust answers are essential to serve the increasing societal demands for actions that reverse current biodiversity trends while maximizing social and economic benefits [30]. We believe this framework contributes to ongoing efforts to expand the scale and investment in biodiversity change assessments. The framework's essential features include the integration of causal models and robust inference practices, including explicit treatment of uncertainty and estimation biases. The detection and attribution framework outlines how our confidence about biodiversity change and its consequences are constrained by our evidence and how we report it. Though work must be done to achieve a full implementation of the framework with clear reporting standards, this could be implemented, for example, by the work of an *ad hoc* Detection and Attribution Working Group of the Group on Earth Observations Biodiversity Observation Network. We believe that the field of biodiversity change science can meet this challenge given the urgency of the need and the recent rapid and ongoing advances in the observation technologies, computational tools and inferential methods required for rapidly detecting and understanding biodiversity change.

## Data Availability

This article has no additional data.
